# Intraoperative detection of parathyroid glands by autofluorescence identification using image-based system: report of 15 cases

**DOI:** 10.1186/s13256-021-03009-8

**Published:** 2021-08-09

**Authors:** Maksym Gorobeiko, Andrii Dinets

**Affiliations:** 1 Department of Surgery, Taras Shevchenko National University of Kyiv, Demiїvska 13, Ukraine, Kyiv, 03039 Ukraine; 2 Department of Surgery, Verum Expert Clinic, Kyiv, Ukraine

**Keywords:** Parathyroid autofluorescence, Hypoparathyroidism, Near-infrared fluorescence, Parathyroid identification, Case report

## Abstract

**Background:**

A detection of parathyroid glands by the evaluation of their autofluorescence in the near-infrared spectrum is considered as a promising tool in addition to their visual verification. The aim of this study was to evaluate the role of near-infrared autofluorescence application by using two different image-based systems for the identification of parathyroid glands during surgery of thyroid and parathyroid benign and malignant lesions.

**Methods:**

Evaluation of near-infrared autofluorescence was performed in 15 patients by using two different image-based systems equipped with a near-infrared laser camera. Intravenous injection of fluorophore indocyanine green was used for the enhancement of near-infrared autofluorescence signal.

**Results:**

Normal parathyroid glands were identified and mobilized after visual inspection in 12 (80%) patients, which was confirmed by near-infrared autofluorescence evaluation. Confident recognition of parathyroid glands by near-infrared autofluorescence signal and their subsequent distinction from lymph nodes was achieved in two (13%) patients with prior surgery for papillary thyroid carcinoma. In one (7%) case, parathyroid gland was identified as fragments of tissue within the postoperative scarring area by near-infrared autofluorescence evaluation, but not by visual inspection. A less intensive near-infrared autofluorescence signal was detected in the parathyroid gland owing to unintentional excision in one (7%) case. Better signal intensity from parathyroid glands was noticed after changes of the near-infrared camera in Fluobeam 800 image-based system in position to an angle of approximately 45–65° in relation to area of interest in all cases as compared with holding straight on the parathyroid gland. Fluobeam LX demonstrated a good near-infrared autofluorescence signal without any specific changes in the camera angle. Thyroid carcinoma demonstrated low-intensity signal in the case of invasion to thyroid capsule. No fluorescent signal was identified from metastatic, or from normal, lymph nodes.

**Conclusions:**

The application of near-infrared autofluorescence imaging is considered as a useful, but additional, tool for the visual assessment of parathyroid gland in the case of primary neck exploration. The utility of near-infrared autofluorescence imaging for parathyroid detection is increased in the case of repeated surgical intervention owing to increased risk of unintentional parathyroid removal as well as for discrimination of parathyroids from the lymph nodes in cases of thyroid malignancy.

## Background

Surgical management of benign and malignant thyroid neoplasms can be complicated by recurrent laryngeal nerve (RLN) damage and parathyroid gland injury. Also, the risk is even higher for injury of parathyroid glands in the case of neck dissection, which is a routine part of thyroid cancer surgical treatment [1]. RLN palsy could be prevented by the application of intraoperative neuromonitoring, whereas identification and preservation of parathyroid glands are mainly under visual verification by the surgeon [2]. It is worth to mention that visual identification of parathyroid glands is easily performed by experienced endocrine surgeons in patients without prior surgery of the central neck compartment [3, 4]. However anatomical location of parathyroid glands is different in the case of surgical interventions for recurrent thyroid cancer or hyperparathyroidism as well as in the case of multiglandular parathyroid gland disease or intrathyroidal localization or other anatomical variations [5–8]. The parathyroid adenoma site is preoperatively identified by 99mTc-sestamibi scintigraphy or computed tomography (CT) scan [6]. Parathyroid hormone (PTH) measurement within 45–120 minutes after the surgery is an indicator of both complete removal of the parathyroid adenoma and postoperative hypoparathyroidism [8, 9]. In contrast, discrimination of metastatic lymph nodes from parathyroid glands in patients with recurrent thyroid cancer is a clinical challenge, associated with a high risk for parathyroid damage [1].

Considerable research has been conducted to establish a diagnostic method for intraoperative mapping of the parathyroid glands, and attempts have been made to address the utility of parathyroid visualization in the near-infrared spectrum [10]. Detection of parathyroid glands by the evaluation of their autofluorescence in the near-infrared (NIR) spectrum is considered as a promising tool in addition to their visual verification, which was introduced by Paras *et al*. in 2011 and considered as a fluorescence-guided surgery (FGS) [1, 11]. The parathyroid gland exhibits autofluorescence in NIR spectrum (NIRAF) of 800–820 nm with a stronger signal as compared with adjacent tissues [4]. This unique feature of parathyroid gland to have a NIRAF was also described in both vascularized and devascularized, and in unintentionally removed, parathyroid gland [1]. Currently, NIRAF is detected by image-based systems to be equipped with the NIR laser camera and console to adjust the NIR signal. FGS studies also attempted to evaluate the utility of NIRAF in combination with a contrast agent. The most common contrast fluorophore to be applied for parathyroid NIRAF evaluation is indocyanine green (ICG). According to published data, the use of such image-based systems is associated with high sensitivity and specificity, but considered as an additional tool for the visual identification of the parathyroid glands by endocrine surgeon [2]. Although detection of autofluorescence of parathyroid gland is becoming more frequent in endocrine surgery centers, there is a controversy for the clinical utility of NIRAF with or without contrast dye enhancement.

The aim of this study was to evaluate the role of NIRAF application by using two different image-based systems for the identification of parathyroid glands during surgery of thyroid and parathyroid benign and malignant neoplasms.

## Methods

Evaluation of the NIRAF was performed in 15 patients who underwent surgical treatment for thyroid and parathyroid neoplasms at the Department of Surgery, Verum Expert Clinic (Kyiv, Ukraine). The study was approved by the local ethical committee. Preoperative assessment of patients included hormonal investigations, clinical blood tests, clinical chemistry, and serum Ca^2+^ as well as evaluation of relevant medical, family, and psychosocial history or genetic information. Thyroid gland was evaluated by ultrasonography in all patients. The thyroid nodules were evaluated by fine-needle aspiration biopsy (FNAB) followed by cytological evaluation according to Bethesda classification. Measurement of serum Ca^2+^ was performed preoperatively and on the first postoperative day, whereas parathyroid hormone (PTH) was measured on the second day after operation. Serum calcitonin was measured in patients with suspicion of medullary thyroid carcinoma. A capsular dissection technique was applied in all operations. During thyroid surgery, all parathyroid glands were identified and mobilized; both recurrent laryngeal nerves were visualized. In the case of parathyroid devascularization, we kept the gland in the area of its primary anatomic localization and avoided parathyroid removal with subsequent autotransplantation in neck muscles. Autotransplantation was performed only for the unintentionally resected parathyroid gland. Organ-preserving operations for patients with thyroid cancer were performed owing to incidental discovery of malignant neoplasm on histopathological evaluation and without preoperative signs of malignancy. Completion thyroidectomy was not performed in the case of thyroid cancer diagnosis of well-differentiated thyroid microcarcinoma (less than 1 cm in the largest diameter). All patients with well-differentiated thyroid microcarcinoma were administrated suppressive thyroid stimulating hormone (TSH) therapy and regular surveillance. The diagnosis was confirmed by the histopathological evaluation of formalin-fixed and paraffin-embedded tissue sections according to World Health Organization (WHO) classification of endocrine tumors [12].

During the operation, the visual identification of parathyroid glands was performed by the surgeon, followed by analyses of the operation field by using one of the available image-based systems. The conformations of visually identified parathyroid glands, as well as other NIRAF signals, were considered for further surgical decisions. NIRAF was evaluated by using the image-based system Fluobeam-800 or Fluobeam LX (Fluoptics, France) equipped with an NIR laser camera, console to adjust NIR signal, and monitor with a touchscreen. NIRAF signal was evaluated after the operation light was turned off. Intravenous injection of fluorophore ICG was used for the enhancement of NIRAF signal. ICG angiography of parathyroid glands was evaluated 1–2 minutes after intravenous injection. Parathyroid NIRAF was evaluated by the manual holding of NIR camera at a distance of 20 cm over the operation field. No quantitative measurements were applied.

## Results

**Fig. 1:**
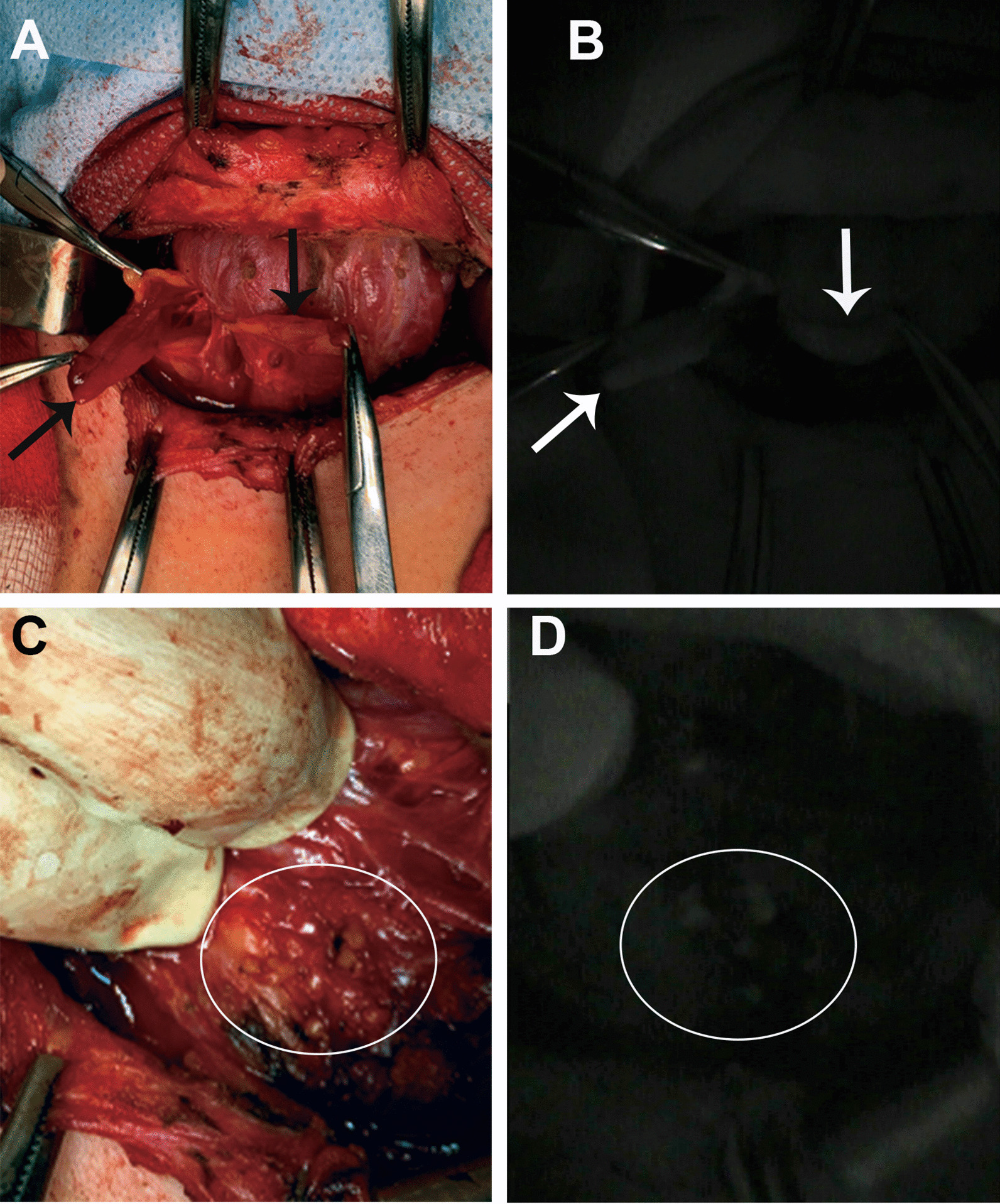
Illustration of intraoperative parathyroid glands (case 15). **A** Photograph of parathyroid adenomas of right upper and lower parathyroid glands in the patient with recurrent primary hyperparathyroidism (indicated with arrows). **B** Photograph of the right parathyroid adenomas showing autofluorescence in near-infrared light (indicated with the arrow). **C** Normal left lower parathyroid gland is presented as fragments of tissue after surgical interventions on the left side (indicated with circle). **D** Photograph of autofluorescence signal of fragmented left lower parathyroid in near-infrared light (indicated with circle)

There were ten (67%) females and five (33%) males with a mean age of 44.5 years (range 27–77 years). Thyroid cancer was diagnosed in nine (60%) patients: classic variant of papillary thyroid carcinoma (PTC) in five (33%) cases, follicular variant of PTC in four (30%) cases, medullary thyroid carcinoma (MTC) in one (7%) case. Benign lesions were constituted by follicular thyroid adenoma (FTA) in two (13%) cases, diffuse toxic goiter (DTG) in one (7%) case, thyroid toxic adenoma in one (7%) case, and parathyroid adenoma (PA) in two (13%) cases. Reoperation was performed in one case of hyperparathyroidism (case 15). Chief-cell parathyroid adenomas were identified in cases 14 and 15. The weight of PTA was 250 mg in case 14, whereas in case 15 two PTAs were 1300 mg and 300 mg (Fig. 1A). All clinical characteristics of the cohort are summarized in Table 1. The analyses of parathyroid identification after the application of two different image-based systems are presented in Table 2.

**Table 1:** Clinical characteristics of study cohort

Patient ID	Sex	Age at operation, years, mean	FNAB class	Operation	Diagnosis	Histopathological features	Tumor size, cm	Tumor foci, *n* =	Location of tumor foci	Preoperative Ca^2+^, mmol/l	Postoperative Ca^2+^, mmol/l	Postoperative PTH, pg/ml
1	M	41	6	TTE, CND, bilateral LND	PTC	Classic variant	1.8	6	Bilateral	1.27	1.08	4.6
2	M	38	6	TTE, CND	PTC	Follicular variant	1.5	1	Unilateral	1.17	1.14	28.9
3	M	56	6	TTE, CND, right LND	PTC	Classic variant, microcarcinoma	0.5	3	Bilateral	1.16	1.18	19.3
4	F	30	5	HTE right, CND	PTC	Classic variant, microcarcinoma	0.5	1	Unilateral	1.31	1.09	31.1
5	F	55	2	HTE right	PTC	Classic variant, microcarcinoma	0.4	1	Unilateral	1.2	1.2	46.2
6	M	77	2	TTE	PTC	Classic variant, microcarcinoma	0.2	1	Unilateral	1.2	1.14	37.7
7	M	38	5	TTE, CND, bilateral LND	PTC	Follicular variant, microcarcinoma	0.4	2	Unilateral	1.3	1.15	4.6
8	F	41	6	TTE, CND, lateral LND	PTC	Follicular variant, microcarcinoma	0.4	3	Bilateral	1.17	1.13	7.0
9	F	34	6	TTE, CND	MTC	Microcarcinoma	0.5	1	Unilateral	1.19	1.17	4.6
10	F	40	3	HTE left	NG, toxic adenoma	n/a	3.5	n/a	n/a	1.21	1.17	n/a
11	F	45	4	TTE	FTA	n/a	3.0	n/a	n/a	1.28	1.06	20.9
12	F	34	4	HTE right	FTA	n/a	3.53	n/a	n/a	1.18	1.18	35.4
13	F	57	n/a	TTE	DTG	n/a	n/a	n/a	n/a	1.28	1.09	73.9
14	F	55	1	Right upper PTE, TTE	PTA, MNG	n/a	n/a	n/a	n/a	1.36	1.18	21.2
15	F	27	n/a	Right PTE	PTA	n/a	n/a	n/a	n/a	1.5	1.22	44

F, female; M, male; FNAB, fine-needle aspiration biopsy class by Bethesda classification; TTE, total thyroidectomy; CND, central compartment neck dissection; LND, lateral neck dissection; HTE, hemithyroidectomy; PTE, parathyroidectomy; PTC, papillary thyroid carcinoma; MTC, medullary thyroid carcinoma; NG, nodular goiter; FTA, follicular thyroid adenoma; DTG, diffuse toxic goiter; PTA, parathyroid adenoma; MNG, multinodular goiter

**Table 2:** Comparison of visual parathyroid glands detection with autofluorescence detection by using image-based system

Patient ID	Age of patient	Diagnosis	Autofluorescence detection methods	Model of image-based system	Correspondence of visual detection of parathyroids with fluorescence image
1	41	PTC	NIR camera only	Fluobeam 800	No
2	38	PTC	NIR camera +ICG	Fluobeam 800	No
3	56	PTC	NIR camera+ICG	Fluobeam 800	Yes
4	30	PTC	NIR camera only	Fluobeam 800	Yes
5	55	PTC	NIR camera +ICG	Fluobeam LX	Yes
6	77	PTC	NIR camera+ICG	Fluobeam LX	Yes
7	38	PTC	NIR camera only	Fluobeam LX	Yes
8	41	PTC	NIR camera only	Fluobeam LX	Yes
9	34	MTC	NIR camera only	Fluobeam LX	Yes
10	40	NG toxic adenoma	NIR camera only	Fluobeam 800	Yes
11	45	FTA	NIR camera+ICG	Fluobeam LX	Yes
12	34	FTA	NIR camera+ICG	Fluobeam LX	Yes
13	57	DTG	NIR camera only	Fluobeam LX	Yes
14	55	PTA, MNG	NIR camera+ICG	Fluobeam 800	Yes
15	27	PTA	NIR camera only	Fluobeam LX	No

NIR, near infrared; ICG, indocyanine green; PTC, papillary thyroid carcinoma; MTC, medullary thyroid carcinoma; NG, nodular goiter; FTA, follicular thyroid adenoma; DTG, diffuse toxic goiter; PTA, parathyroid adenoma; MNG, multinodular goiter

**Fig. 2:**
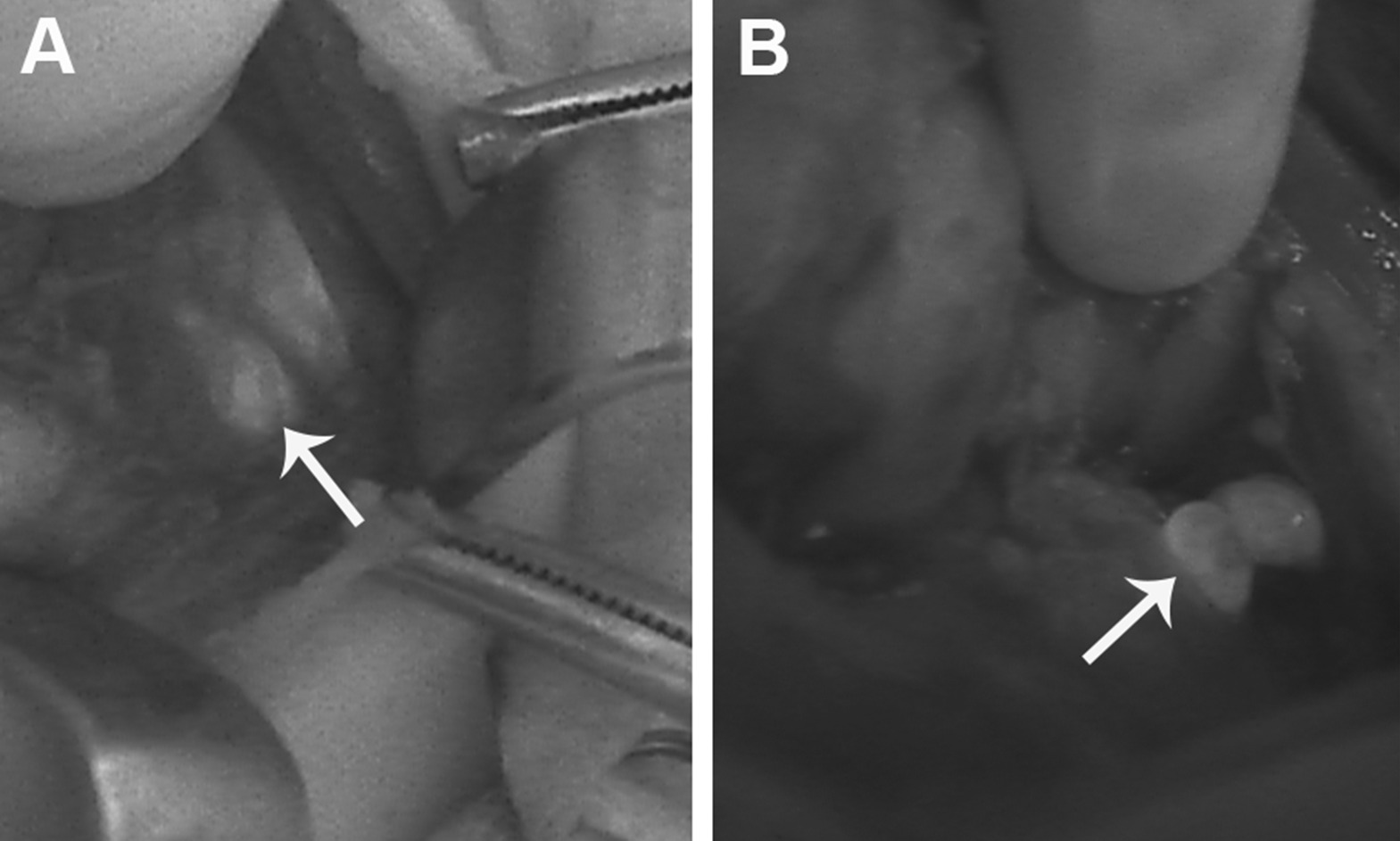
**Illustration of normal parathyroid gland in near-infrared light**. **A** Autofluorescence signal in the well-vascularized parathyroid gland (indicated with arrows). **B** Autofluorescence signal is detected in the parathyroid gland (indicated with arrows) with compromised blood supply

Normal parathyroid glands were identified and mobilized after the visual inspection in 12 (80%) cases, which was confirmed by NIRAF evaluation (Fig. 2). The distinct identification of parathyroid gland by visual inspection was controversial in three cases (cases 1, 2, 15). In cases 1 and 2, the visual identification of lower parathyroid glands was problematic during the neck dissection for PTC. The application of an image-based tool for NIRAF evaluation was associated with a confident recognition of both lower parathyroid glands and their discrimination from lymph nodes in cases 1 and 2. In the patient with PTA (case 15), there was a recurrence of hyperparathyroidism in both right parathyroid glands, and the patient had a history of removal of upper left parathyroid adenoma 1 year prior to disease relapse in another endocrine surgery center. The parathyroid adenomas were identified with their further confirmation by NIRAF (Fig. 1A, B). Although the tissue scarring was not advanced, the identification of remnant parathyroid glands was not fully achieved by visual assessment. However, the evaluation of NIRAF resulted in the distinct identification of the remnant parathyroid gland (Fig. 1C, D). It is worth mentioning that the left lower parathyroid gland in case 15 was in the area of previous parathyroid surgery for hyperparathyroidism, and we were not able to identify it at visual inspection. However, evaluation of NIRAF confirmed that the left lower parathyroid gland was present in the area of the left lower thyroid pole as tissue fragments (Fig. 1C).

In the patient with PTA (case 14), ICG was injected after the adenoma was identified at visual assessment and confirmed by NIRAF without ICG. In this case, we noted equal distribution of the NIRAF signal in both thyroid gland and PTA. However, 15–20 minutes after ICG injection, thyroid tissue exhibited stronger signal as compared with PTA with a less intense NIRAF signal. This observation indicates that NIRAF in combination with ICG might be useful for the identification of the hidden PTA in other anatomical locations, but in a limited timeline of up to 20 minutes after exposure to the contrast agent.

A weak signal was detected from the normal thyroid gland as well as from the thyroid and parathyroid benign tumors by using fluorescence tool with and without ICG. Less intensive NIRAF signal was also detected in cases 7, 8, and 9, indicating possible devascularization of parathyroid glands (Fig. 2B), which was subsequently confirmed by low postoperative PTH (Table 2). Also, a less intensive NIRAF signal was detected in the parathyroid gland owing to unintentional excision (case 1). The ICG enhanced NIRAF detection was performed in seven (47%) cases after mobilization of parathyroid glands. All parathyroid glands were identified visually prior to confirmation by NIRAF with and without ICG enhancement. The NIRAF signal from the parathyroid glands was stronger after the ICG application as compared with non-contrast cases.

To our surprise, we noticed a better signal intensity from the parathyroid glands after the changes of the NIR camera in Fluobeam 800 in position to an angle of approximately 45–65° in relation to the area of interest in all cases as compared with holding straight on the parathyroid gland. By doing such a maneuver, we confirmed parathyroid glands in the location next to the thyroid gland as well as in the thymus tissue. In contrast, Fluobeam LX demonstrated a good NIRAF signal without any specific changes of the camera angle, which is associated with better confidence in the distinction of NIRAF signal by the surgical team.

In this case series, we also evaluated NIR signals in various thyroid malignancies. Thyroid carcinoma demonstrated low-intensity signal in the case of invasion to the thyroid capsule. The signal from thyroid cancers was different as compared with normal thyroid, but still less intensive as compared with the parathyroid gland. Low-intensity fluorescence signal was detected in two patients with thyroid cancer invasion to adjacent neck muscles after ICG application. There was no identified fluorescent signal from metastatic or from normal lymph nodes.

## Discussion

In this study, we evaluated two different image-based systems for NIRAF detection with and without ICG application for identification of parathyroid gland in patients with thyroid and parathyroid tumors. This case report is a demonstration of FGS and the utility of NIRAF as an additional tool for the verification of parathyroid glands during operations for thyroid and parathyroid lesions.

Similar to other authors, we have detected all parathyroid glands in most of the cases by visual recognition using anatomical landmarks [4, 7, 8, 13]. Still, we were not confident about parathyroid glands locations in two cases (13%), which is in line with the findings from the larger cohorts [4, 13].

We reported a low rate of unintentional parathyroid excisions, which was also demonstrated in other FGS studies with and without NIRAF application. Despite the low rate of unintentional parathyroid removal, we reported postoperative hypoparathyroidism, which was probably associated with an impaired blood supply of parathyroid glands. This finding is in agreement with Papavramidis *et al*., who also reported a lower rate, but not the complete reduction of postoperative hypoparathyroidism in the case of NIRAF evaluation.

In this study, we also showed a problem for lower parathyroid gland visual identification during the central neck dissection for thyroid malignant neoplasms. Due to the high risk of metastases in the central neck compartment, it is crucial to not mix up potentially metastatic lymph nodes with parathyroid glands [4]. Another problem that we have also demonstrated was the identification of the parathyroid gland in the central neck compartment after the previous surgical interventions. In such a situation, NIRAF evaluation is an additional and useful tool for the preservation of the parathyroid glands from unintentional removal [14].

In our opinion, NIRAF evaluation is not associated with a 100% successful identification of the parathyroid glands, because other adjacent tissues could also exhibit a signal, making a strong background. On the other hand, the parathyroid gland does not always provide a strong NIRAF signal. During NIRAF evaluation, we also noticed a signal from the thyroid tissues as well as from a thyroid toxic adenoma. Such signal patterns from the other tissues have also been shown by Cui *et al*. and Idogawa *et al*. suggesting “White out” and “Black out” patterns at NIRAF evaluation [15, 16]. In our study, “White out” was considered as a situation of the presence of stronger fluorescence signal from the thyroid gland than from the parathyroid gland. In contrast, “Black out” pattern was described as an absence of NIRAF signal. Taken together, strong background fluorescence, as well as the absence of the signal effects, might be considered as limitation factors for NIRAF evaluation.

The evaluation of two different models of image-based systems showed that the location of NIR camera position with an angle of approximately 45–65° in relation to the area of interest was associated with better visualization of the parathyroid gland on the screen (model Fluobeam 800). Such changes in the exposure angle in this device might be associated with different localization of the parathyroid gland, and therefore such a positioning of the NIR beam was matched with the larger number in fluorophore substrates in the parathyroid gland. This specific finding was not described before, and we consider it as a possible useful clinical tip while performing NIRAF evaluation. In contrast, the newer image-based system Fluobeam LX showed a good NIRAF signal without any specific changes in the camera position. Still, such a conclusion was made on a small study cohort, which is a limitation.

Our study may have more limitations. For example, our results were obtained from using two image-based systems, whereas other devices may show different data on this study cohort. Still, according to published reports, all other available image-based systems shared similar features for NIARF detection, except the fiber probe-based system, namely PTeye, which required gentle interaction between the probe tip and target tissues [1].

We have also demonstrated the similar utility of NIRAF evaluation with ICG enhancement as compared with NIRAF alone. However, the application of ICG remains controversial. According to published series, ICG enhancement might overwhelm the autofluorescence in the case of missing parathyroid gland owing to ICG incorporation by the thyroid gland, making a strong background signal [4, 17]. Such a situation could occur in cases of different anatomical locations of the parathyroid gland, in cases of multiglandular disease, or after previous operation in the central compartment of the neck. In contrast, Cui *et al*. suggested NIRAF accompanied by an ICG enhancement as an important tool for detection of parathyroid adenoma for secondary hyperparathyroidism [16].

## Conclusions

To summarize, our findings demonstrated that FGS with NIRAF evaluation is associated with better results of identification and preservation of parathyroid glands. The application of NIRAF imaging is considered as a useful, but additional, tool for the visual assessment of the parathyroid gland in the case of primary neck exploration. However, the utility of NIRAF imaging for parathyroid detection is increased in the case of repeated surgical intervention, due to the changes in anatomical landmarks and increased risk of unintentional parathyroid removal as well as for distinction of lymph nodes in cases of thyroid malignancy. Application of ICG is not associated with better identification of parathyroid glands, and specific features of the NIR camera position should be considered while using different image-based systems.

## Data Availability

All data generated or analyzed during this study are included in this published article.
